# Development of a chicken 5 K microarray targeted towards immune function

**DOI:** 10.1186/1471-2164-7-49

**Published:** 2006-03-13

**Authors:** Jacqueline Smith, David Speed, Paul M Hocking, Richard T Talbot, Winfried GJ Degen, Virgil EJC Schijns, Elizabeth J Glass, David W Burt

**Affiliations:** 1 Division of Genetics and Genomics, Roslin Institute, Roslin (Edinburgh), Midlothian, EH25 9PS, UK; 2 Ark-Genomics, Roslin Institute, Roslin (Edinburgh), Midlothian, EH25 9PS, UK; 3 Intervet International B.V., Dept. of Vaccine Technology and Immunology R&D, P.O. Box 31, 5830 AA, Boxmeer, The Netherlands

## Abstract

**Background:**

The development of microarray resources for the chicken is an important step in being able to profile gene expression changes occurring in birds in response to different challenges and stimuli. The creation of an immune-related array is highly valuable in determining the host immune response in relation to infection with a wide variety of bacterial and viral diseases.

**Results:**

Here we report the development of chicken immune-related cDNA libraries and the subsequent construction of a microarray containing 5190 elements (in duplicate). Clones on the array originate from tissues known to contain high levels of cells related to the immune system, namely Bursa, Peyers patch, thymus and spleen. Represented on the array are genes that are known to cluster with existing chicken ESTs as well as genes that are unique to our libraries. Some of these genes have no known homologies and represent novel genes in the chicken collection. A series of reference genes (ie. genes of known immune function) are also present on the array. Functional annotation data is also provided for as many of the genes on the array as is possible.

**Conclusion:**

Six new chicken immune cDNA libraries have been created and nearly 10,000 sequences submitted to GenBank [GenBank: AM063043-AM071350; AM071520-AM072286; AM075249-AM075607]. A 5 K immune-related array has been developed from these libraries. Individual clones and arrays are available from the ARK-Genomics resource centre.

## Background

In recent years, the tools available to the field of chicken genomics have increased greatly. Detailed genetic and physical maps have been constructed [1], as well as BAC contig maps [2,3] and a radiation hybrid panel [4]. There is also a substantial EST collection [5], SNP database and many full-length cDNAs have been sequenced. The development of these resources has culminated with the recent publication of the chicken draft sequence [6]. The chicken can now be regarded as an important model organism for use in comparative genomics, residing in a potentially informative position in the evolutionary ladder. The chicken is also an extremely useful model for developmental biologists and geneticists as well as being a commercially important species.

The latest tools being developed for the chicken are microarrays. There are several small tissue-specific arrays being used by individual labs. These include an intestinal array (3,072 clones) [7], a macrophage-specific array (4,906 clones) [8], a lymphocyte array (3,011 clones) [9] and an 11 K array based on genes found in heart progenitor cells [10]. A 13 K genome-wide array is also available from ARK-Genomics [11] (Roslin, UK) and from the Fred Hutchinson Cancer Research Centre (Seattle, USA) [12]. We have designed a 5 K immune-related array created from libraries developed from tissue (Bursa, spleen, Peyers patch, thymus) from birds which were previously inoculated with a combination of different vaccines to various common avian diseases including bacterial, protozoa and virus disease-causing organisms (*E. coli*, Newcastle Disease Virus (NDV), Infectious Bursal Disease Virus (IBDV), coccidiosis, Marek's Disease (MD) and salmonella). The tissues we chose are highly representative of T and B cell populations and were used in order to optimise the numbers of immunologically – related genes that would be present in our libraries. Many known immune genes that have been recently identified in the chicken EST collections [13] have also been added to the array. This array provides a valuable, cost-effective resource for the investigation of immunological gene expression. It has been created from a pool of stimulated immune tissues and contains genes that represent a wide spectrum of immune functions as well as previously unidentified sequences. Each gene on the array is also functionally annotated as much as possible. Gene ontology [14] data and Blast [15] information is provided for each clone, where that information is available.

## Results and discussion

### Construction of the array

Six immune-related libraries were specifically developed for the construction of a 5 K chicken array. Immune tissue from birds inoculated with different vaccine regimes (see Methods) was used to develop two standard libraries. These both underwent two rounds of normalization, thus providing us with six libraries. Initially, 10,173 clones were randomly chosen from the libraries for sequencing. The number chosen from each library depended on the titre (colonies/microlitre) of that particular library. The 10,173 clones that were sequenced were searched for poor quality sequence (<100 bp after removal of vector, repeats etc.) and unwanted Blast homologies, as described in the Methods section. The numbers of high quality sequences (9,434 – which have been submitted to Genbank) from each library are shown in Table 1. Cluster analysis was then undertaken, which resulted in the grouping of clones from which we would choose the 5,000 that were to be represented on the array.

### Genes on the array

The clones on the array are derived from custom-made immune-related chicken cDNA libraries. Libraries developed from tissue from Bursa, spleen and Peyers patch were our representative 'B cell' libraries, and libraries developed from thymus were so-called 'T cell' libraries (the names 'B and T cell libraries' are used purely for ease of reference and in no way indicate that the libraries consist of pure cell populations). Clones from both standard and normalized libraries are present on the array. One clone representing each of the 3,811 clusters is included on the array, along with a random selection of singleton clones (1,067). The sequence of each of the clones was also subjected to a Blast search of the SwissProt and TREMBL databases and the highest hit to each sequence was reported. Searches were carried out at a stringency of 1e^-10 ^(this relatively low stringency was to ensure that we identified as many immune homologies as possible). Chicken immune genes have a relatively low level of sequence conservation with mammals, hence the lower stringency used in these searches). We wanted to ensure that certain genes were also represented on the array as 'reference' genes. This included a range of known immune-related genes for which a clone was already available – either from the existing EST databases [12] or from our novel libraries. Various cytokines, chemokines, cell surface antigens, receptors and MHC molecules were all included (Table 2). The expression profile of genes of unknown function can thus be compared with the profiles of these genes whose roles are known. Standard array controls were also spotted on the array, including various spot report buffers (positive and negative controls for the Cy3 and Cy5 dyes), salmon sperm DNA, calf thymus DNA, bovine genomic DNA (negative controls), chicken genomic DNA, gamma actin and GAPDH (positive controls). Each clone is represented in duplicate.

### Analysis of the immune clones

All the sequences of the clones on the array were subject to Blast homology searches against the SwissProt and TREMBL databases using a cut-off value of 1e^-10^. Using this means of detection, many known immune-related molecules were identified, including cytokines, interferons, interleukins, transcription factors, receptors, cell differentiation antigens, MHC molecules and genes for proteins belonging to the TOLL receptor pathway. Proteins homologous to hypothetical human proteins and mouse cDNAs were also identified.

Sequences, which gave no Blast homology to anything in the nucleotide or protein databases, accounted for about 38% of the clones. Either the search parameters were too stringent to identify these genes or the chicken sequence was sufficiently divergent to be undetectable in a standard Blast search. This is a common feature of immune-related genes, and it is often very difficult to identify such genes by sequence homology to mammalian homologues. Some of these sequences may also represent non-conserved 3' UTR regions of genes. This set of clones may also include genes that have never been identified before and are not represented in the sequence databases. Further, more detailed analysis of these sequences can sometimes help elucidate the nature of the gene in question. Protein sequences can be predicted from the EST nucleotide sequence using programs such as ESTscan [[16] and [17]], which takes in to account sequencing errors and thus potential frame-shift mutations which are often present when there is only one EST sequence available for study. Conserved motifs and domains can then sometimes be identified for example, using the Pfam database [18], which is a large collection of multiple sequence alignments and hidden Markov models covering many common protein domains and families. PSI-Blast searches can also help identify to which type of family a gene will belong.

During clustering analysis, our 10,000 immune sequences were aligned with 398,000 existing chicken ESTs. This highlighted 3,845 clusters that contained one or more sequence from our immune libraries and 1,959 singleton clones. This analysis also identified 40 novel clusters that only contained sequences from our new libraries. Upon Blast analysis, 7 of these clusters were found to represent known chicken genes (initially appearing unique as they aligned to a different part of the gene sequence from existing ESTs), 18 showed homology to genes in other species and 15 clusters proved to have no known homology to anything currently in the databases. At the time, we searched against 398,000 existing chicken ESTs. Now however, there are currently 550,510 chicken ESTs in the databases (dbEST release 080505). A current search has shown that 9 of our sequences are indeed still unique to our libraries and have no known identifiable homologue, although two of the sequences do show some similarity to two predicted chicken sequences (AM065333 and the hypothetical protein XP_429359; AM065802 and the predicted P114-RHO-GEF protein XP_418249). Eight of these sequences are identifiable in the whole genome sequence, as shown in Table 3.

### Gene ontology (GO) annotations

In order to try and elucidate the function of the genes on the array further, we tried to assign as much annotation to the sequences as possible. GO annotations were assigned to some sequences after searching the GGI and UMIST databases [19], while other annotation was derived from hits to orthologous human sequences from the ENSEMBL [20] and GENSCAN [21] databases, as described in the 'methods' section. Having annotation derived from orthologous human genes means that cross-species comparisons between chicken and human array data may be possible. A search of the ENSEMBL database provided information on 2,292 GO-term associations, the GGGI database 1,542 and GENSCAN 566, while the UMIST full-length cDNA database provided a further 365 annotations. The sequences on the array cover a total of 227 GO terms, with 73% of all the sequences having at least one GO entry assigned to it. The available annotation for the array sequences is broken down as follows: 52% of genes have a 'cellular component' term assigned, 60% have 'molecular function' and 56% of sequences have the 'biological process' described. 83% of all the genes on the array have some kind of gene description and after searching each sequence against the sequences in the Ensembl chicken genome collection (July 2005 genebuild [22]), 78% of sequences were found to have a known chromosomal location. Now that all these sequences have been added to GenBank and thus have an accession number which can be directly linked into the ENSEMBL databases (work currently underway), obtaining comprehensive, up-to-date annotation data will become much easier.

A file showing the complete annotation for all the sequences on the array is available as supplementary material (Additional file 1). However, Additional file 2 provides an overview of the broad functional classes that are represented by the genes on the array. These are based on more general GO annotations derived from the GO-slims database at EBI, and allow us an insight into the different classes of genes present on the array without having to look at detailed functional annotation for each individual gene.

Annotation is also available for some (9,137) of the ESTs in the UMIST collection. By comparing the relevant GO slims [23] terms for the sequences in this collection with those present on our array, we are able to see which types of genes appear to be enriched in our set, compared with a larger, more general collection of EST sequences. As can be seen (shown in bold) in Additional file 2, certain classes of gene appear to be more highly represented. For instance, genes involved in protein transport are more abundant in our set of clones, as are those involved in the response to stimulus. This is consistent with our attempts to pre-select for higher numbers of genes involved in the immune system.

### Quality of the array

**Figure 1:**
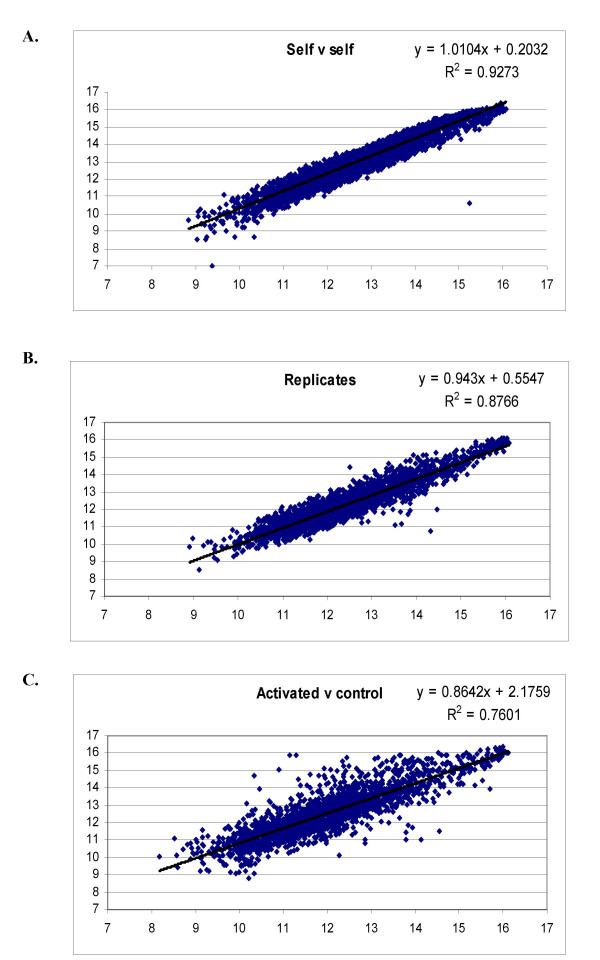
Scatter plots showing the variance between A). self/self hybridisation B). two biological replicates and C). a control sample compared with an activated sample. Very little spread is seen with the self/self hybridisation and between the two replicates, as would be expected. However, differences in gene expression can be seen between the activated and control samples.

To assess the quality of the array, various hybridization comparisons were undertaken. Three different conditions were addressed: 1). self v self 2). biological replicate A v biological replicate B and 3). Control sample v activated sample. Dye swap experiments were also carried out for conditions 2 and 3. The 'self' sample was a reference RNA consisting of a pool of various chicken lung samples. The biological replicates were lung samples from two 6-week-old chickens that had not been treated or challenged in any way. In the third group of hybridisations, the 'control' sample was from a similarly, untreated bird and the 'activated' sample was obtained from the lungs of a bird that had been challenged with the avian influenza strain H9N2 five days previously. The graphs in Fig 1 show the tight correlation between self/self (R^2 ^= 0.9273) and between replicates (R^2 ^= 0.8766), whereas a much higher level of variance is seen when an activated sample is compared against a control (R^2 ^= 0.7601).

**Figure 2:**
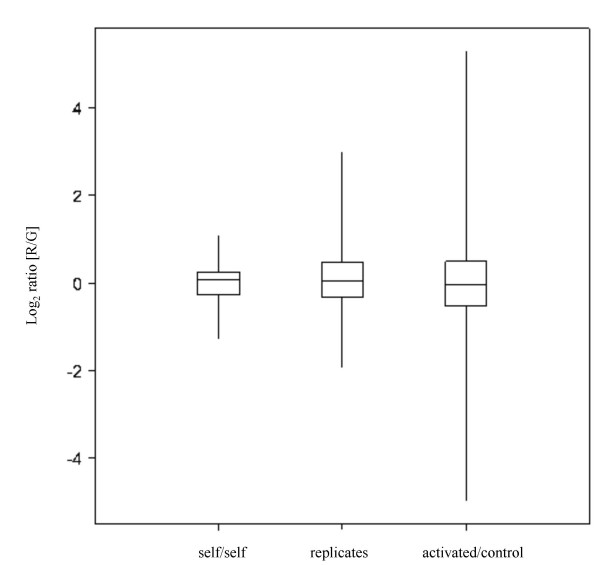
Box plots showing the variance between self/self hybridisation, two biological replicates and a control sample compared with an activated sample. Boxes represent the interquartile range from 25–75%, with the median marked. Outliers to this range are also shown.

The boxplots in Fig 2 also demonstrate the differing variances between the comparisons. The greatest variance is shown for the activated animals compared with the controls as would be expected. Regression analysis for each of the data sets confirm the increased variance with correlation coefficients of r = 0.872 for activated samples, r = 0.936 for replicate samples and r = 0.963 for self/self sample data sets.

### Using the array

This array is available from the Ark-Genomics resource facility at Roslin Institute, providing an immune-focused array which, for anyone interested in immune-research, offers a much more cost-effective and time-saving platform for gene expression experiments, instead of using the large oligo arrays which have thousands more genes, many of which will be of no interest. Analysis of data is also thus much easier and far less time-consuming. Information on the array has been deposited in ArrayExpress (Accession: A-MEXP-307) [[24] and [25]] (Additional file 1) and very soon all the sequences will be submitted to the Ensembl database with links to all the GO annotation information in the GOA database [26].

## Conclusion

We have constructed a 5 K chicken cDNA microarray, which is highly selected for genes expressed in tissues which have an immune function. This targeted array contains enough widely-expressed genes (whose expression won't be changing) to enable good normalization, as well as containing numerous known immune genes (from our novel libraries and from existing EST collections). The array also contains many genes with as yet unknown homology and function as well as a few novel genes which are specific to the libraries from which the array was created. These genes of unknown function could well have a role in either the adaptive or innate immune response, and thus provide a valuable resource for analysis of gene expression changes occurring in birds that have been subject to immune challenge. The array has been proven to provide highly reproducible results and is now available to the chicken/microarray community as a whole.

## Methods

### Sample collection

Eight groups of 38 chickens (3-week-old) were vaccinated with two different vaccine regimes. The eight groups were males and females of a commercial line of hybrid broiler (Ross 306, Aviagen, Newbridge, Midlothian, UK) and layer (Lohman Brown, Lohmann Tierzucht, Cuxhaven Germany) chicks given one of the two vaccination schemes. Group 1 were given vaccines for *E. coli *(0.5 ml in left breast muscle), ND and IBDV (0.5 ml in right breast muscle) formulated in alum-gel and oil-based immuno-potentiators. Intramuscular injections were given to ensure that all the birds were given an equal dose. Group 2 vaccines consisted of Paracox 8 [*Eimeria *sp.] (0.1 ml in drinking water), Nobilis Rismavac-CA126 [MD] (0.2 ml intramuscularly in leg) and Salenovac [*S. enteritidis*] (0.5 ml intramuscularly in leg). Tissue samples were obtained (unvaccinated); 5 hr, 24 hr, 72 hr and 7 days post vaccination. Samples from groups of 5 birds were pooled. Tissues collected were Bursa, spleen, Peyers patch and thymus. Tissue from Bursa, spleen and Peyers patch were pooled to make the 'B-cell' libraries and the thymus tissue was used to construct the 'T-cell' libraries. The tissues and time points chosen were in order to try and maximise the number of immune-related transcripts, including those which may only be expressed transiently. All experimental protocols were authorized under the UK Animals (Scientific Procedures) Act, 1986.

### Library construction

Six libraries were constructed at Incyte Genomics (Palo Alto, CA): a standard and 2 normalized Bursa/spleen/Peyers patch libraries and a standard and 2 normalized thymus libraries. cDNA synthesis was initiated using an oligo (dT) primer, using methylated C in the first strand synthesis reaction. Following this first strand reaction, double-stranded cDNA was blunted, ligated to *NotI *adapters, digested with *EcoRI*, size-selected, and cloned into the *NotI *and *EcoRI *compatible sites of a custom modified MCS of the pBluescript (KS+) vector. Normalization was done in two rounds using conditions adapted from [[27] and [28]] except that a significantly longer re-annealing hybridization was used. Around 10,000 clones were then sequenced at the Sanger Institute according to their protocols. Using the T7 primer, sequence was generated from the 5' end of each clone by the dideoxy chain termination method using an ABI 3700 sequence analyser (Applied Biosystems, Foster City, CA).

### EST sequence analysis

Bioinformatic analysis commenced with 10,173 sequences. After eliminating poor quality sequence and repeats, 9,434 of these sequences remained after screening with phred [29], RepeatMasker [30], Crossmatch [31] and XNUN [32]. Certain unwanted sequences were then identified after using the Blast algorithm [[33] and [34]] and screening the results for specific keywords. These included 'ribosomal', 'mitochondrial', 'Newcastle', 'Mareks', 'Eimeria', 'Salmonella' and 'E. coli'. 8,154 sequences passed these criteria. These sequences were then clustered against the existing UMIST and EMBL chicken EST sequences using TIGR's clustering tool, tgicl [35]. This resulted in 3,845 clusters which contained one or more sequence from our libraries and 1,959 singletons. The following clones were chosen for inclusion on the array: 3,770 cluster representatives, 1,067 singletons and 157 reference immune genes: 93 clones from the UMIST collection, 41 from our immune libraries, 21 clones from the Delaware set [36] and 2 clones courtesy of R. Zoorob (CNRS, France) (Table 2).

### Construction of the array

The immune array was constructed from 4994 chicken EST clones plus 196 control elements (landing lights (positional controls), GAPDH, gamma actin, salmon sperm DNA, calf thymus DNA, chicken and bovine genomic DNA and a variety of spotting buffers). Plasmid DNA was prepared using MagAttract 96 Miniprep chemistry on a Biorobot 8000 platform (Qiagen Ltd., Crawley, UK), and the cDNA inserts were amplified using CGATTAAGTTGGGTAACGC (fwd) and CAATTTCACACAGGAAACAG (rev) in 50 ul reactions using 1 ul of DNA as a template. Amplified DNA was purified by Multiscreen 384 well PCR purification plates (Millipore, Watford, UK) on a Multiprobe II liquid handling platform (Perkin Elmer, Beaconsfield, UK) and the reactions confirmed by agarose gel electrophoresis and quantified by Picogreen assay (Molecular Probes, Invitrogen, Paisley, UK) on a Flouroskan Ascent flourescent plate reader (Thermo Life Science, Basingstoke, UK). DNA was resuspended to 150 ng/ul in spot buffer (150 mM Sodium phosphate, 0.01% SDS) before being spotted in duplicate on to amino-silane coated slides (CMT-GAPSII, Corning, Schiphol-Rijk, The Netherlands) using a Biorobotics MicroGrid II spotter (Genomic Solutions, Huntingdon, UK). Slides were then treated using succinic anhydride and 1-methyl-2-pyrrolidinone (Sigma, Poole, UK) to block unbound amino groups, followed by a wash in 95°C MilliQ water before hybridisation.

### RNA preparation and labelling

Total RNA was isolated from lung tissue using a Trizol extraction according to the manufacturer's protocol (Invitrogen, Paisley, UK) and subsequently purified using the RNeasy Midi RNA Purification kit (Qiagen Ltd., Crawley, UK). RNA concentration was determined spectrophotometrically and RNA quality was determined using an Agilent 2100 Bioanalyser (Agilent Technologies, Waldbronn, Germany). Cy3 or Cy5 was incorporated into each sample using the Fairplay labelling kit (Stratagene, La Jolla, CA) and the labelled cDNA cleaned-up after passage through DyeEx columns (Qiagen Ltd., Crawley, UK). Labelling efficiency was determined by running 0.5 μl of each sample on a 1% agarose gel and measuring the intensity of fluorescence on a GeneTac LS IV scanner (Genomic Solutions, Huntingdon, UK).

### Hybridizations

Microarray hybridizations were carried out overnight using a GeneTAC automated hybridization system [37] (Genomic Solutions, Huntingdon, UK). Hybridizations (125 μl) were carried out in Genomic Solutions hybridization solution (Cat. no. RP#0025) in a stepped hybridization: 55°C for 3 hr, 50°C for 3 hr and then 45°C for 12 hr. Slides were then washed in Genomic Solutions wash buffers (Cat. nos. CS#0038, CS#0039 and CS#0040). Upon removal from the hybridization stations, slides were washed for 1 min in Post-Wash buffer (CS#0040) and a further minute in isopropanol, followed by centrifugation at 1000 rpm for 6 min. Dried slides were scanned in a Scanarray 5000 scanner (GSI Lumonics, Rugby, UK) fitted with Cy3 and Cy5 filters.

### Data analysis

To indicate the suitability of the new array to discriminate the differences in the experimental treatments, hybridizations comparing samples with controls and controls with controls were performed. Control (vehicle treated) animals were compared with immunologically challenged animals (activated slides) and control animals were also compared with other control individuals (replicate slides). The same animal was also compared with itself (self/self). Each comparison was completed in duplicate and with a dye flip. Dye-swaps are carried out in order to deal with any residual dye-bias remaining after labelling. However, this is generally not a problem, due to the indirect labelling method employed. Data was extracted from the slide using Bluefuse software (BlueGnome, Cambridge, UK). Features with poor confidence information (confidence <0.30, flagged D and E) were eliminated from the analysis. M v A plots [where M = log_2 _(Cy5/Cy3) and A = 1/2*(log_2_(Cy5) + log_2_(Cy3)] of the data for each slide (data not shown) were suitably linear to require only a simple global normalisation of the data. Data from slides of similar treatments was pooled and a boxplot produced for each comparison (Genstat v8.1, VSN International Ltd., Hemel Hempstead, Herts, UK).

### Databases and sequence sources

Ensembl and Genscan predicted genes/peptide sequences for the chicken genome assembly (March 2004) were downloaded from the Ensembl database using Ensmart or the UCSC table browser [38]. Chicken EST sequences were downloaded from the TIGR *Gallus gallus *gene index (GGGI) [release 10.0] [[39] and [40]]. Chicken full-length cDNA sequences were downloaded from the UMIST www site (Sept 2004). Ensembl predicted peptide sequences for the human genome assembly (May 2004) were downloaded from the Ensembl database using Ensmart or the UCSC table browser.

### Mapping array probes to chicken ESTs, cDNAs, genes and genome

Unique ESTs used to create the immune array were mapped to chicken cDNAs, ESTs, genes or the chicken genome assembly using NCBI Blastn (version 2.2.11). Identity was defined with > 95% sequence identity over 100-bp and then taking the top-scoring match to each EST to provide a unique sequence assignment. All repeats and low-complexity sequences were masked using RepeatMasker (version 3.1.0).

### Definition of Gene Ontology terms and Gene Descriptions for array probes

Gene Ontology (GO) annotations [41] were all based on database hits in sequence similarity searches using Blastn. GO annotations were automatically transferred from these database records to the array probe entries. GO annotations were available for GGGI and UMIST EST/cDNA sequences. For chicken Ensembl or Genscan gene predictions, GO annotations were based on orthologous human peptide sequences. Orthologues were defined based on two cycles of Blastp between human and chicken proteins. An E_value cut off of less than 10^-4^, with the subject and query databases swapped between runs. By comparing E_values mutually best proteins pairs were selected as orthologues. When E_values were equal, bits score and sequence coverage were used as tiebreakers to select the top hit. For each array probe associated GO terms and a unique gene description was transferred from the orthologous database record. Finally a Perl script was used to create a non-redundant set of probe to GO records.

### Frequency of GO and GO-Slim terms

GO terms (version 3.2.16) were downloaded from the Gene Ontology www site. More general GO terms were assigned using GoaSlim_map (June 2005) available from the GOA www site at EBI. The GO-Slim terms allowed us to estimate e.g. the frequency of array probes associated with the biological process *Metabolism *(*GO:0008152*).

### Data processing

Perl scripts (version 5.8.5) and SQL were used throughout to manipulate and filter data sets.

## Authors' contributions

JS contributed to the design of the array, carried out the microarray experiments and drafted the manuscript. DS carried out quality control, clustering and BLAST searches of the DNA sequences. PH was responsible for tissue sample collection. RTT statistically analysed the microarray data. WGJD and VEJCS were involved in experimental design. EJG contributed to the array design. DWB carried out all the bioinformatics involved in establishing GO annotations. All authors read and approved the final manuscript.

## Supplementary Material

Additional File 1 The file supp_mat.xls is an excel file which contains annotation information on the sequences present on the immune array and is available as supplementary material. An ArrayExpress  file is available under accession number A-MEXP-307. Click here for file

Additional File 2 The file supp_mat_2.xls is an excel file and contains a summary of functional groups present on the array (GO slims). Percentages are calculated as a fraction of the total number of classes represented within one functional description. For example, 42.4% of all the genes are involved in some kind of physiological process. Of these, 14.3% are involved in transport, with 10.2% of these genes being specifically involved in electron transport. This breakdown of functional classes is compared to those represented by 9,137 of the ESTs in the UMIST collection (data available from ). Entries shown in bold define the GO classifications that appear to be enriched in the sequences represented on the immune array compared with this subset of the UMIST chicken ESTs. Click here for file

## Figures and Tables

**Table 1:** Clones sequenced from each library.

**Library**	**No. of clones**
Chicken immune 1 ('B cell' standard)	975
Chicken immune 2 ('B cell' normalized 1)	2,394
Chicken immune 3 ('B cell' normalized 2)	1,600
Chicken immune 4 ('T cell' standard)	2,563
Chicken immune 5 ('T cell' normalized 1)	918
Chicken immune 6 ('T cell' normalized 2)	984

**Table 2:** List of known immune genes added as reference genes to the array.

**Gene**	**EST**	**Clone ID**	**Accession no.**
**AH221**	**CTN2_C0000858f10.q1kT7SCF**	**C0000858F10**	AM064266
AH294/RANTES	603404971F1	C0000737M17	BU397782
**β2 microglobulin**	**CTst_C0000869a17.q1kT7SCF**	**C0000869A17**	AM068376
**BAFF**	**CBN1_C0000466j11.q1kT7SCF**	**C0000467J11**	AM066201
BMP10	604156553F1	C0003869J14	BU210183
BMP2	603213309F1	C0003763A3	BU444424
BMP4	603363891F1	C0000429F23	BU473912
BMP6	603604307F1	C0003811M1	BU287807
BMP7	603500540F1	C0000717O9	BU333004
BMP8A	603956728F1	603956728F1	BU425800
CC chemokine receptor 6	603508559F1	C0002794E15	BU267610
CC CKR 11	603367511F1	C0000439E3	BU465158
CC LARC/MIP-3A	603534015F1	C0002806N3	BU398190
**cCAF**	**CBN1_C0000465h11.q1kT7SCF**	**C0000465H11**	AM065832
CD135 antigen	603812446F1	C0001334K11	BU376898
CD137	pat.pk0038.d7.f	C0004737E4	AI980851
CD153	pat.pk0072.b5.f	C0004738K22	AI982035
CD154	603535227F1	C0001006F7	BU398104
**CD18**	**CBN1_C0000468e11.q1kT7SCF**	**C0000468E11**	AM066422
CD2	pgn1c.pk014.i9	pk014I9	CB017050
CD200	pat.pk0062.c8.f	C0004738C17	AI981679
CD226	pat.pk0020.d12	C0004739G22	AI980296
**CD28**	**CTst_C0000892d20.q1kT7SCF**	**C0000892D20**	AM070143
**CD3**	**C0001679M3_G02_008.AB1**	**C0001679M3**	AM070515
CD36	603543789F1	C0001028A23	BU311037
CD4	pk017a12	pk017a12	CB017654
CD40L	pgm2n.pk009.d11	pk009d11	BM488880
CD44-like	603745662F1	C0003894K15	BU253134
CD45	603767294F1	C0003827K23	BU446679
CD59	603212850F1	C0000363D13	BU447971
CD63 antigen	603783352F1	C0001268G14	BU243877
CD7	pat.pk0040.d6.f	C0004737K4	AI981043
**CD79A**	**CBst_CHK02000039f07.q1kT7SCF**	**CHK0200003F7**	AM071949
**CD8**	**CTst_C0000877k01.q1kT7SCF**	**C0000877K1**	AM069615
**CD82**	**CTst_C0000892l24.q1kT7SCF**	**C0000892L24**	AM070329
CD83 antigen	603771889F1	C0001238B24	BU457418
**CD84**	**CTN1_C0000798o19.q1kT7SCF**	**C0000798O19**	AM070961
**CD98 light chain**	**CBN1_C0000465c24.q1kT7SCF**	**C0000465C24**	AM065739
Chemokine receptor like 2	603764351F1	C0001219F13	BU444213
Chicken cytokine	pat.pk0050.e9.f	C0004737J11	AI981311
CHIR-A	603478533F1	C0003884A9	BU359209
**CHIR-B**	**CBst_CHK02000039l03.q1kT7SCF**	**CHK0200003L3**	AM072078
cMGF	pat.pk0060.h1.f	C0004737P22	AI981598
**Complement C3**	**CBN1_C0000468j22.q1kT7SCF**	**C0000468J22**	AM066546
Complement C4	603811612F1	C0001332G3	BU376477
Complement C7	603668434F1	C0001140D9	BU416108
Complement C8α	603782386F1	C000164L21	BU242118
complement H	603735023F1	C0001154N6	BU295434
complement receptor 1	603819479F1	C0001351N20	BU268132
**Complement1**	**CBN2_C0000485f23.q1kT7SCF**	**C0000485F23**	AM068133
Cremp	603114782F1	C0003743C21	BU126768
**C-type lectin**	**HFU603551466C18**	**C0004763C18**	AM063354
CX 3C chemokine receptor 1	603949695F1	C0003852N23	BU204148
**CXC-R4**	**CTst_C0000878f17.q1kT7SCF**	**C0000878F17**	AM069849
Cytokine like protein 17	603773283F1	C0001242E21	BU459791
Cytokine receptor like 9	603472805F1	C0000591J1	BU477689
**Death receptor 6**	**CBN1_C0000466l11.q1kT7SCF**	**C0000467L11**	AM066244
DSL-1	603321647F1	C0000418M11	BU239031
EMAP II	603364164F1	C0003776P16	BU475067
ephrin type A receptor 2	603121949F1	C0000241A13	BU133519
FAS antigen	603737578F1	C0001181L19	BU300974
**FASL decoy receptor 3**	**CTN2_C0000856k13.q1kT7SCF**	**C0000856K13**	AM064070
**GATA-3**	**CTN2_C0000858a24.q1kT7SCF**	**C0000858A24**	AM064179
GDF10	603530236F1	C0000994G10	BU351257
GDF8	603775823F1	C0001248N6	BU458566
GDF9	603741256F1	C0001166O13	BU300398
glycoprotein 130	603369157F1	C0002739A15	BU460413
GMCSF	CF258055	CF258055	CF258055
HCC1	pat.pk0059.g4.f	pk059g4	AW061438
ICSBP	603568552F1	C0001037G14	BU383423
IFNα	603486811F1	C0000622F3	BU319434
IFNα/β-R2	603783234F1	C0001268C9	BU243612
IFNγ	603766180F1	C0003825O20	BU444142
IFNγR2	603606133F1	C0001120B10	BU294744
IFP35	603123028F1	C0000243H7	BU135331
**Ig light chain VJC region**	**C0000914E7_C03_018.AB1**	**C0000914E7**	AM064528
Ig heavy chain VDJ region	603534767F1	C0002807F18	BU398082
IK cytokine	603368212F1	C0000440H3	BU460192
IL-10	CF258071	CF258071	CF258071
IL11 receptor	603402722F1	C0000518K5	BU250398
IL-12β	603603708F1	603603708F1	BU291084
IL12-p35	603761859F1	C0002846F16	BU474924
IL-13R2	603519773F1	C0000972A19	BU341330
IL-15	603102514F1	C0002655L4	BU202444
IL-16	603130176F1	C0002702H20	BU114872
IL17 receptor	603211483F1	C0000350E22	BU448712
IL-18	603508766F1	C0002794D18	BU271029
IL-1β	603217760F1	C0003766G15	BU455380
IL-2	pat.pk022.e2	pk022e2	AI980311
IL20 receptor	603591538F1	C0001088K9	BU241765
IL-2Rα	pat.pk0012.h3	pk0012h3	AI980106
**IL-2Rγ**	**CBN1_C0000360j15.q1kT7SCF**	**C0000360J15**	AM064841
IL-4	603772775F1	ChEST708f19	BU450270
Il-4R	603490820F1	603490820F1	BU324362
IL-6	pat.pk0076.f2.f	C0004739G21	AI982185
Interleukin enhancer binding factor 3	603322645F1	C0000420J12	BU239448
IRAK2	603831145F1	ChEST821j11	BU435261
IRAK4	603208981F1	ChEST185a21	BU441365
IRF1	603960463F1	C0002900N15	BU418343
**IRF10**	**CBN1_C0000360l15.q1kT7SCF**	**C0000360L15**	AM064884
IRF2	604146465F1	C0003862A18	BU438609
**IRF3**	**CTst_C0000892j09.q1kT7SCF**	**C0000892J9**	AM070266
**IRF4**	**6O4_B10_077**	**C0000885O4**	AM072251
IRF5	pat.pk0067.c5.f	pk067c5	AI981854
IRF6	603111427F1	C0000188F7	BU109331
**JSC**	**CTst_C0000878m23.q1kT7SCF**	**C0000878M23**	AM069996
K60	603470605F1	C0002774I2	BU479398
lymphotactin	603733847F1	C0001151N12	BU300469
MCSF1-receptor	603220574F1	C0000383C19	BU432910
**MDV vIL-8**	**CBst_C0000222p17.q1kT7SCF**	**C0000222P17**	AM071831
**MHC class I**	**CBst_CHK02000039o05.q1kT7SCF**	**CHK0200003O5**	AM072147
**MHC class I minor**	**CTst_C0000873a15.q1kT7SCF**	**C0000873A15**	AM068728
**MHC class II beta**	**HFU603551341A11**	**C0004763A11**	AM063247
MIF	604141521F1	C0001517I5	BU438017
MX	603775783F1	C0001248E22	BU457953
NKL	603539011F1	C0001016E4	BU309556
N-pac	604157079F1	604157079F1	BU210048
NRAMP1	pk013p5	pk013p5	BI394251
NRAMP2	603953027F1	C0001451E8	BU203948
OCIF	603157972F1	C0000333O17	BU410189
opioid receptor sigma 1	603341826F1	C0003775C8	BU254440
Orphan chemokine receptor	603234142F1	C0000403C12	BU418544
PIT54	603150061F1	C0000313I16	BU126277
platelet activating receptor	pat.pk0002.b12	C0004739A2	AI979750
PRC1	603475588F1	603475588F1	BU355757
**prostaglandin synthase**	**CBN1_C0000466j02.q1kT7SCF**	**C0000467J2**	AM066193
Regulator of cytokinesis 1	603475588F1	C0000598P22	BU355757
**RING3**	**CBst_C0000222p04.q1kT7SCF**	**C0000222P4**	AM071819
**SCA-2**	**CTN1_C0000853f13.q1kT7SCF**	**C0000853F13**	AM071100
SCYa13	603534566F1	603534566F1	BU397023
SCYA4	603742061F1	C0001168I15	BU299262
SIGIRR	603321436F1	C0002934H10	BU240159
SOCS1	603758706F1	C0003823K20/L1	BU218362
SOCS2	603322984F1	603322984F1	BU239208
SOCS5	603492126F1	C0000636C7	BU326390
STAT1	603957345F1	ChEST927i11	BU425993
STAT2	pat.pk0027.a6.f	C0004737C3	AI980571
**STAT5b**	**CBst_C0000222j02.q1kT7SCF**	**C0000222J2**	AM071688
TAP2	603732809F1	C0000758A3	BU298074
**Tapasin**	**CTst_C0000873d22.q1kT7SCF**	**C0000873D22**	AM068799
TARC	pat.pk0031.f10.f	C0004737K13	AI980713
T-bet	pgn1c.pk013.h8	pgn1cpk013.h8	CB016768
**Tcell receptor α**	**CBst_CHK02000039p10.q1kT7SCF**	**CHK0200003P10**	AM072172
**Tcell receptor β**	**UEB603581072O18**	**C0004765O18**	AM063532
**Tcell receptor γ**	**CTst_C0000878m08.q1kT7SCF**	**C0000878M8**	AM069981
**Tcell receptor ζ**	**CTst_C0000874j17.q1kT7SCF**	**C0000874J17**	AM069278
TGFβ	603758578F1	C0003823K20	BU215243
**Thymosin beta 4**	**ODP603945810C04**	**C0004766C4**	AM063804
TLR1/6/10	603760940F1	C0001211O10	BU471724
TLR2	603588755F1	C0001081M13	BU374739
TLR3	603781018F1	C0001261D6	BU242827
TLR4	603470778F1	C0002774L20	BU475859
TLR5	603230983F1	C0000395E22	BU420247
TLR7/8/9	603160284F2	C0002711G22	BU435893
TRAF1	pat.pk0072.d3.f	C0004738M6	AI982046
TRAF2	603217872F1	C0003766O18	BU455745
**TRAF5**	**CTst_C0000877n08.q1kT7SCF**	**C0000877N8**	AM069687

The genes in bold come from the immune libraries described in this paper

**Table 3:** Genomic location of unique chicken ESTs as identified by the University of Santa Cruz Blat site

**Clone**	**GenBank Accession No.**	**Genomic location**
CBN1_C0000465p01.q1kT7	AM065989	chr15: 5562143–5562781
CBN1_C0000464a07.q1kT7	AM065333	chr11_random: 637442–638117
CBN1_C0000465g01.q1kT7	AM065802	chr28: 3396525–3399444
CBN2_C0000485f09.q1kT7SCF	AM068122	chr7: 1319368–1319989
CBst_C0000222i16.q1kT7SCF	AM071684	no hit
CTN2_C0000856o09.q1kT7	AM064132	chr17: 9342673–9343272
CBN1_C0000360j04.q1kT7	AM064831	chr21: 2494538–2494813
CBN1_C0000360n18.q1kT7	AM064932	chr1: 172893324–172893990
CBN1_C0000463a02.q1kT7	AM064982	chr4: 50741781–50742197
